# Bridging Functions and Values: Advancing Wetland Ecosystem Service Assessment Through Use of a Service Capacity Index

**DOI:** 10.1007/s13157-025-02028-1

**Published:** 2026-03-04

**Authors:** William J. Kleindl, Sarah P. Church, Kai C. Rains, Mark C. Rains, Eric D. Stein, Morgan K. Suddreth

**Affiliations:** 1 Dept of Land Resources and Environmental Sciences, Montana State University, 334 Leon Johnson Hall, Bozeman, MT 59717 USA; 2 Dept Earth Sciences, Montana State University, 226 Traphagen Hall, Bozeman, MT 59717 USA; 3 School of Geosciences, University of South Florida, Tampa, FL 33620 USA; 4 Southern California Coastal Water Research Project, Costa Mesa, CA 92626 USA

**Keywords:** Wetland function, Wetland ecosystem service, Assessment, Regulatory, Compensatory mitigation

## Abstract

Wetland service or values are often assumed to occur at a comparable level to wetland function (i.e., higher functioning wetlands provide more services). However, services are seldom directly evaluated because of the lack of structured assessment approaches. For wetland ecosystem service to be effectively incorporated in regulatory and monitoring programs, there needs to be parsimonious assessment tools that can be applied systematically, repeatably, and rapidly. This approach begins by disentangling existing terminology and clearly defining all the terms necessary to support the assessment of ecosystem services (ES). We provide context to illustrate how these terms have been variously employed in wetland science and policy, and follow with a conceptual framework for a rapid wetland ES assessment tool that is implemented as a module to existing rapid wetland function or condition assessment tools. We employ a *service capacity index* (SCI) that builds on existing concepts of functional capacity indices (FCIs) by incorporating the *opportunity* for beneficiaries to avail themselves of the source and sink ecological products provided by wetlands. We argue that if the goal of assessment is to meet compensatory mitigation requirements of unavoidable loss of wetland service, an SCI is an appropriate complement to an FCI. We illustrate the application of the proposed approach using Montana floodplain wetlands as an example.

## Introduction

Wetland assessment tools have been developed in the U.S. to support federal, state, and tribal monitoring and assessment programs, as well as to meet the regulatory obligations of the Clean Water Act (CWA) and laws enacted by state and local governments. They are often used to support ambient monitoring programs that report on the condition of aquatic resources, to identify impaired waters (e.g., CWA Sect. 303(d)), and to support analyses associated with authorizing unavoidable impacts on aquatic systems (e.g., CWA Sect. 404(b)(1)). Other tools have been tailored to meet the resource management needs of agencies (e.g., BLM 2001) or to fulfill other agency missions (e.g., USDA 2012). A specific need typically defines the most appropriate assessment tool (Stein et al. 2009). Therefore, there are almost as many assessment tools as specific needs; for example, there are as many as 12 rapid assessment tools available for use on the relatively narrow category of floodplain wetlands in the headwaters of the Missouri and Yellowstone Rivers in Montana (U.S.) alone (Kleindl et al. 2024).

Most wetland assessment tools have been motivated by the primary objective of protecting habitats, or ecological structure or function, as opposed to the human benefits derived from healthy ecosystems. This is despite the often-stated policy or regulatory objective of protecting both ecology and the human use of the environment. Although the scientific literature documents many of the historical advancements in wetland concepts, much of the development and application of assessment methods has occurred outside academia and within governmental agencies (see, for example, Fennessy et al. 2007, and references therein). The decentralized and iterative aspect of this development has led to a suite of specific terms and concepts. Terms such as functions, conditions, values, and services are often used incorrectly and/or interchangeably. In academic settings, confusing terms and concepts are generally resolved as the literature advances. However, incorrect and/or interchangeable uses can become codified in regulatory frameworks and linger in the lexicon. This has created confusion in and debate over the common language of assessment and monitoring, with even the meaning of the term rapid assessment engendering vigorous debate (Fennessy et al. 2007; Kleindl et al. 2010).

**Fig. 1:**
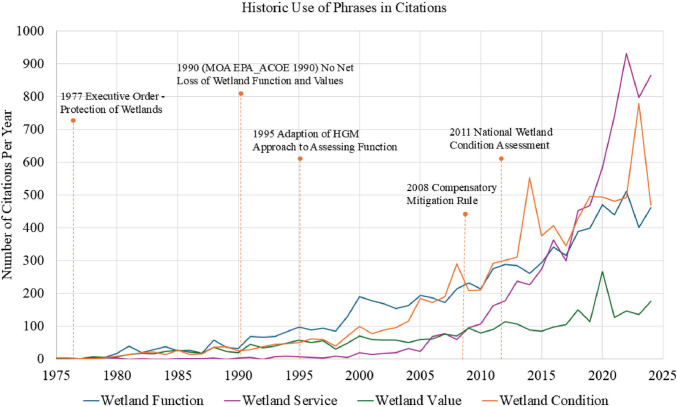
Timeline of notable wetland policy adoptions in the U.S. and trendlines depicting the frequency of keyword phrases in the literature (1975 −2024) using keyword phrases related to the Ecosystem Services concept that is a recent assessment trend (i.e., *“wetland function”* OR *“wetland ecosystem function,”* and replaced *function* for *service*, *value*, and *condition*). Source database: Digital Science’s Dimensions (Digital Science 2024), which includes more than 155 million publications

More recently, there has been renewed attention on the ecosystem services (ES) that wetlands provide to maintain human well-being (Fig. 1). This has been driven by an increased recognition and appreciation of the important services that healthy ecosystems provide for people. This, in turn, has motivated the development of policy directives within the US that require greater attention to ES. Early policy directives from the US federal government, such as the 2015 memorandum from the Obama Administration requiring federal agencies to factor ES into federal planning and decision-making (OWH 2015) and the Biden Executive Order 14072, requiring guidance to include ES in forest management decisions, helped motivate the development of ES assessment tools within the federal government, such as EPA’s Final Goods and Services (USEPA 2017) and the National Ecosystem Services Classification System (USEPA 2020a). For wetlands, the 1990 memorandum of agreement was signed between the U.S. Environmental Protection Agency (USEPA) and the U.S. Army Corps of Engineers (USACE) to ensure that there is no net loss (NNL) of wetland function and values (USEPA 1990) was a foundational guidance in wetland assessment of wetland services, leading to novel tools emerging to assess those ES (e.g., Stelk and Christie 2014, USEPA 2020a, 2020b). Much of this work builds upon wetland ES efforts in Europe (see Maltby 2009; Haines-Young and Potschin-Young 2018). Yet, it recognized that the complexity of language, as well as the need for standardized measurement and reporting, are barriers to integrating ES policy (Keenan et al. 2019). This is especially true where a confusion over wetland assessment terminology persists, and explicit linkages between assessments of functions (or conditions) and ES are lacking. Here, we review the history of assessment language, clarify the definitions of key terms, and provide a framework to link the function (or condition) assessment tools of today to the ES assessment tools of tomorrow.

## History of Wetland Assessment Terminology

Wetland assessments have stemmed from regulatory needs, which are driven by evolving sociocultural values as reflected in executive orders and statutes. As management and science approaches evolved to account for these sociocultural values, new terms emerged. The following is a brief history of these terms and their integration into the wetland assessment lexicon.

### Wetland Capacity

As early as 1977, an executive order by U.S. President Carter instructed federal agencies to minimize the loss of wetlands (GPO 1977). This and other policy directives, such as the CWA of 1977, motivated the USEPA to develop a program to control pollution of the nation’s wetlands. In those policy directives, there are requirements to account for project impacts on aquatic resources, and thus began the development of wetland classification (Cowardin et al. 1979), delineation (Environment Laboratory 1987), refined wetland mapping (Tiner 1990; Dahl 1991), and wetland assessment (USFWS 1980). The U.S. Fish and Wildlife Service’s Habitat Evaluation Procedure (HEP: USFWS 1980) is an early example of a method to assess ecological impacts developed to support the National Environmental Protection Act (NEPA: U.S.C. 1969). A focus of HEP is to assess the existing wetland habitat relative to the concept of *capacity*–optimal habitat condition stemming from carrying capacity (sensu MacArthur and Wilson 1967). Although the term “capacity” persisted, end-users felt that HEP was too time-consuming and too narrowly focused on fish and wildlife to use in a regulatory setting (NRC 1995).

### Wetland Function, Effectiveness, Opportunity, and Social Significance

In 1983, the Federal Highway Wetland Assessment (FHWA) was developed as a nationwide wetland permitting tool for Federal highway projects (Adamus and Stockwell 1983). It was intended to address a broad range of wetland functions to assist regulators in the CWA, Sect. 404(b)(1) permit review process (GPO 1986). Later, FHWA was updated and renamed to Wetland Evaluation Technique (WET) (Adamus et al. 1991). WET tightly links ecological processes to ‘*goods and services*.’ This paired phrase has a history dating back to Adam Smith and is a common term in contracting language, referring to items that are commodities in an economy (Smith 1778; Insider 2025). WET refers to goods and services as ecological products that solely benefit humans and defines *wetland functions* as biophysical processes that support only human needs. WET additionally incorporated the concepts of *effectiveness*, *opportunity*, and *social significance*. In their conceptual framework, a wetland has the *capacity* (*effectiveness*) to perform a function that benefits human needs (e.g., stormwater detention). Yet, if there is no *opportunity* for that process to occur (e.g., no stormwater enters the wetland), then the function does not exist, and the wetland will, therefore, have a low *social significance* (NRC 1995). Therefore, using WET, wetlands in undeveloped areas would have low effectiveness, opportunity, and social significance and tend to score low, and in developed areas, even as the system becomes ecologically degraded, wetlands would tend to score high. End-users objected to the counterintuitive results and also expressed frustration that the nationwide approach did not account for regional differences (Novitzki et al. 1997).

### Biological Integrity and Aquatic Condition

On a parallel track, in 1987, the USEPA called for effective methods to assess the ecological health of the Nation’s surface waters (USEPA 1987). To meet this mandate, USEPA developed the Rapid Bioassessment Protocols (RBP: Barbour et al. 1999). RBP incorporated aspects of the Index of Biotic Integrity (IBI: Karr 1986) developed previously to meet the CWA mandate to maintain the chemical, physical, and biological integrity of our Nation’s waters. Karr and Dudley (1981) define *biological integrity* as ‘…balanced, integrated, adaptive community of organisms having a species composition, diversity, and functional organization comparable to the natural habitat of the region.’ Both methods, RBP and IBI, use reference-based data of biotic composition to assess the *aquatic condition*, or the departure from integrity, of a site relative to a regional disturbance gradient. These approaches have been criticized for not being rapid enough to integrate these assessments into decision-making needs (Fennessy et al. 2007; Kleindl et al. 2010).

### Rapid Assessment

To help meet the CWA Sects. 303(d), 305(b), and 404(b)(1) obligations, the USEPA recommends that states and tribes develop rapid assessment methods (Barbour et al. 1999). To meet those recommendations, the USEPA has categorized ecological assessment tools into a three-tiered approach: Tier 1 – landscape assessments, Tier 2 – rapid assessments, and Tier 3 – highly detailed assessments with intensive data collection and analysis (Fennessy et al. 2007). In Tier 2 – rapid assessments, *rapid* assessment is defined as an assessment that can be completed by two people in one-half day in the field and one-half day in the office (Sutula et al. 2006; Fennessy et al. 2007). Tier 2 – rapid assessments are designed to be efficient approaches to providing the data necessary for permitting decision-making and are the most common wetland ecological assessment strategy within the U.S. (Fennessy et al. 2007).

### Wetland Function (revised) and Wetland Value

In 1990, a memorandum of agreement was signed between USEPA and the USACE to ensure that there is NNL of wetland function and values (USEPA 1990). To meet that mandate, the USACE developed the Hydrogeomorphic Approach to Assess Wetland Functions (HGM Approach) (Brinson et al. 1994; Smith et al. 1995). Like RBP and IBI, the HGM Approach is specific to regions and types of wetlands (i.e., HGM classes), and requires reference data collection for calibration (Kusler 2006), and is intended to be *rapid* (Sutula et al. 2006; Fennessy et al. 2007; Kleindl et al. 2010). In the HGM Approach, ecosystem structural attributes are assessed relative to reference-based disturbance gradients and then combined into a measure of divergence from optimal capacity to perform a suite of functions (Brinson et al. 1994). The HGM Approach utilizes *capacity* in the same sense as it was previously used in HEP.

In a significant departure from WET, the HGM Approach decoupled humans from the definition of the word *function*, defining functions as “physical, chemical, and biological processes” (Smith et al. 1995). This use of the term *function* is consistent with definitions used in ecological literature (sensu Odum 1962) and was codified in the 2008 Compensatory Mitigation Rule (GPO 2008). Thus, the HGM Approach requires distinguishing between objective measures of ecosystem structure and the subjective components of values – attributes that are important to humans. Notably, the HGM Approach provided some limited guidance for decision-makers to connect functions to values (Smith et al. 1995), yet offered no way to connect functions to values quantitatively. Common criticisms of the HGM Approach were that it was too conceptually complicated, not rapid, and did not include assessment of values or direct consideration of the public interest review process (Kusler 2006; Fennessy et al. 2007; Kleindl et al. 2010). Additionally, despite the efforts of the HGM Approach to redefine *function* in the assessment literature, a review of 40 assessment tools in use in the late 1990 s by Bartoldus (1999) found that over half used the term *function* as a measure of *value* (i.e., functional-value), which undoubtedly contributed to ongoing miscommunication and confusion (Kusler 2006).

### Wetland Condition (revised)

In 1999, the USEPA began a series of reports to assist States and Tribes in evaluating wetland conditions and the stressors that affect conditions as part of their CWA Sect. 305b water quality reports (USEPA 2001). To facilitate this national effort, USEPA developed the National Aquatic Resource Surveys (USEPA 2013) and began conducting national wetland condition assessments in 2011 (USEPA 2016). This effort inspired the development of several rapid assessment methods (RAMs) across the country (e.g., Collins et al. 2006; USEPA 2011), with many states subsequently using these RAMs to support programs related to CWA Sect. 404. In general, RAMs focused on assessing wetland *condition*, defined as “the ability of an aquatic resource to support and maintain a community of organisms having a species composition, diversity, and functional organization comparable to reference aquatic resources in the region” (Fennessy et al. 2007). Here, Fennessy et al. (2007) defined *condition* in the same way Karr and Dudley (1981) defined *biological integrity*. This definition of wetland *condition* was also codified in the 2008 Compensatory Mitigation Rule (GPO 2008). Developing these tools was a cost-effective response to the more expensive, complex development of the HGM Approach tools. They are rapid, cover a wide range of physiographic regions within each state, and rely on literature and expert opinion to establish reference conditions.

### Wetland Ecosystem Services

From the No Net Loss directive in 1990 to the introduction of the HGM Approach in 1995, there was no official guidance on the use of the terms *functions and values* in wetland programs, and these terms were often used interchangeably in statutes, regulations, and reports (Kusler 2006). However, in the mid-1990s, the concept of *ecosystem services* was defined as the elements of ecosystems that maintain human well-being (Boyd and Banzhaf 2007), gained mainstream recognition through scientific and conceptual advances in the field. The burgeoning concepts of wetland ecosystem services helped provide insight into the confusing concepts of wetland value (e.g., Zedler and Kercher 2005; Wardrop et al. 2011; Turner et al. 2012). The 2008 Compensatory Mitigation Rule, which supersedes the 1990 NNL mandate, states that “compensatory mitigation should be located... where it is most likely to successfully replace lost functions and services” (Ruhl et al. 2009). Ruhl et al. (2009) noted that this vital phrase is likely to gain policy traction only if efficient and reliable methods exist to assess wetland ES. These methods did not exist, which is likely why ecosystem values and services were infrequently included in the assessment approaches.

### Monetized Value

Monetized value builds upon the earlier value definitions (above) and dominates the ecological service literature, which emphasizes that an ES is synonymous with elements of capital (Brown et al. 2007; Jenkins et al. 2010; Young and Loomis 2014). In ES capital flow models, ES produce natural capital (e.g., recreational fishing) that engages with human capital to produce tangible goods (e.g., fishing equipment) and also engages with cultural capital to produce intangible benefits (e.g., enjoyment from a day fishing) (Boyd et al. 2016; Jones et al. 2016). Note that the paired phrase ‘goods and benefits’ used in some literature is the same as ‘goods and services’, which linger in earlier publications and regulatory tools. There is a suite of tools to determine the value of realized goods and benefits (see Loomis et al. 2000; Stelk and Christie 2014; Young and Loomis 2014 for excellent examples). However, these tools require extensive economic models and are consistent with protocols for a Tier 3 – highly detailed assessment approach in the USEPA assessment hierarchy (Fennessy et al. 2007) and, therefore, are not rapid.

## Definitions Moving Forward

Central to developing a wetland ES assessment tool is establishing a strong conceptual connection between the ecological outputs produced by the relationship of wetland structure and processes, and the interaction of those products with the human community (Peterson et al. 2010; Boyd et al. 2016; Van Wensem et al. 2017). For an ES assessment tool to effectively integrate into the existing wetland assessment literature and ultimately into application, we need to incorporate and clearly define the language currently used in the scientific literature, as well as its relationship to regulatory directives designed to meet management needs. We suggest incorporating a selection of the terms introduced above into a conceptual design to ensure consistency with contemporary usage of that language and facilitate its incorporation into regulatory and other assessment programs.

Many assessment approaches employ a functional capacity index (FCI) in their assessment framework (e.g., Smith et al. 1995; Berglund and McEldowney 2008; CWMW 2013). This is an efficiency required to ensure the approach is rapid. For example, measuring the total amount of carbon sequestered in a wetland would require intensive data collection (Tier 3 in USEPA assessment hierarchy; Fennessy et al. 2007), while measuring conditions to infer the capacity of a wetland to sequester carbon requires only minimal data collection (Tier 2 in USEPA assessment hierarchy; Fennessy et al. 2007). FCI calculations are the foundation for determining debit/credit allocations in compensatory mitigation areas where functional-based mitigation is the standard.

Wetland functions produce sink (e.g., carbon sequestration) and source (e.g., dissolved organic carbon) end products, a subset of which may benefit human well-being (Boyd et al. 2016; Kleindl et al. 2018). The quality of these end products is dependent on the capacity of the wetland to produce them, as reflected by the FCI. Further, the degree to which a beneficiary engages with these products informs its contribution to human well-being. This degree of engagement may be characterized by attributes such as the type of beneficiaries affected and their proximity to the wetland. Here, we propose an expansion on the 1995 National Research Council (NRC) definition of opportunity (as defined above), incorporating a measure of beneficiary engagement with the products of wetland processes. Unlike the NRC (1995) definitions, we follow the HGM Approach and argue that wetland functions occur regardless of human benefits; however, the capacity of the wetland to provide those benefits is dependent on both the quality of the ecosystem sink and source products *and* the degree of engagement of the beneficiaries.

We propose using a capacity approach to assess wetland ecosystem service through a *service capacity index* (SCI). We argue that if the goal of the assessment is to meet compensatory mitigation requirements of unavoidable loss of wetland service, an SCI is an appropriate complement to an FCI for determining compensatory mitigation units. Because most assessment tools used by States are Tier 2—rapid assessments (Sutula et al. 2006; Fennessy et al. 2007), the SCI tool must also be rapid. Many states currently have one or more rapid wetland condition/function assessment tools to support their management needs (Kleindl et al. 2024). Adding an additional requirement for a new assessment can create unacceptable additional workload or expertise needs. To minimize this additional effort, we propose a module that interacts with the output of existing wetland condition or function assessment tools (FCI), coupled with the opportunity for benefactors to engage with those functions to calculate the SCI.

**Fig. 2:**
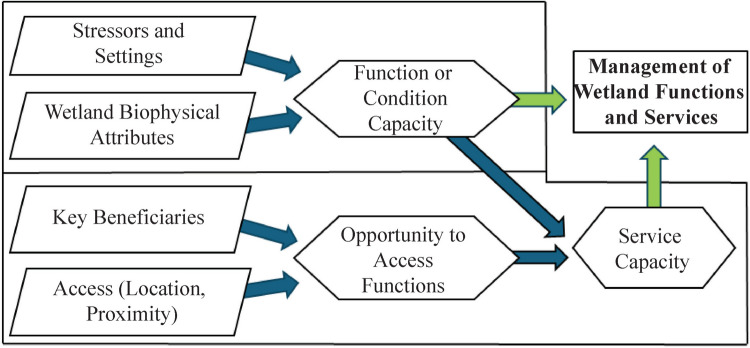
Conceptual framework to connect existing functional assessment tools to the service assessment modules

Following this conceptual approach, we propose using the following definitions (incorporated in Fig. 2):

- *Beneficiaries* – Individuals, groups of people, or organizations that directly use or appreciate an ecological end-product, resulting in a positive or negative impact on their welfare (USEPA 2020a).
- *Functional Capacity Indices* – Functional capacity is the degree to which an area of wetland performs a specific function (Smith et al. 1995). A functional capacity index (FCI) is the ratio of the functional capacity of a wetland of interest under existing conditions to the functional capacity that a wetland exhibits under reference conditions (Smith et al. 1995).
- *Opportunity* – A measure of beneficiary engagement with the end-products of wetland ecological processes.
- *Service Capacity Indices* – A service capacity index (SCI) is the capacity of a wetland to produce ecological end products scaled to the opportunity of the beneficiaries to engage and benefit from those products.

## Conceptual Approach to Integrate Rapid Wetland Function/Condition Assessment with Wetland Ecosystem Services Assessments

Most U.S. wetland assessment approaches that support regulatory obligations of the CWA are biased toward assessing wetland function and condition. For example, within Montana and the four surrounding U.S. intermountain western states, twenty-five ecosystem assessment tools were developed to measure aquatic health, and in a few cases, limited measures of ecological services (Kleindl et al. 2024). Those tools that measure services are often based on the assumption that high-functioning wetlands also provide high-quality services, as seen in Montana’s wetland assessment methodology (Berglund and McEldowney 2008). Others blend functions and services as a residual from earlier definitions that stem from WET, such as Washington State’s wetland ranking protocol (Hruby and Yahnke 2023). There are stand-alone ES assessment tools, such as USEPA’s Rapid Benefit Indicators (USEPA 2020b), but these do not include aspects of wetland functions. Lastly, tools like the Wetland Ecosystem Service Protocol (Adamus 2016) rapidly measure both functions and services, but they are not integrated into existing assessments currently in use.

Here, we propose a conceptual framework, using the refined definitions above, for a rapid wetland ES assessment tool that will serve as a module to accompany existing rapid wetland function or condition assessment tools (Fig. 2). In this conceptual framework, an existing assessment tool is used to calculate an FCI, which accounts for a wetland’s setting and stressors that impact the condition of biophysical attributes. Collectively, these quantify the condition of the ecological sink and source products produced or supported by the wetland through the FCI. A subset of these products supports the well-being of certain beneficiaries. The beneficiaries’ ability to access these ecological products defines a scalable opportunity. The combination of the functional capacity to produce these ecological products and the opportunity to access that capacity defines the ability of the wetland to provide that service, or the service capacity. The functional capacity and the service capacity are now units that meet CWA Sects. 303(d) – identify impaired waters; 305(b) – biannual reporting on aquatic condition; and 404 – credits or debits that can be accounted for in compensatory mitigation as needed.

## Example Application

The following is an example of how the conceptual framework could be applied to assist in meeting CWA Sect. 404 permitting and compensatory mitigation needs. It is not intended as a final assessment model, but rather as an illustrative example of a possible approach to address flood attenuation ecosystem services provided by floodplain wetlands. Floods are among the most common, widespread, and deadly natural disasters in the United States, causing more fatalities annually than other disasters like tornadoes and wildfires (USGS 2017; Endter‐Wada et al. 2018; Gill et al. 2021). Floodplain wetlands play a crucial role in temporarily storing floodwaters, making flood attenuation one of the essential services provided by wetlands, in terms of dollars saved and losses avoided (Acreman and Holden 2013; Watson et al. 2016; Endter‐Wada et al. 2018). For example, at local scales, floodplain wetlands can provide hundreds of thousands of dollars in flood mitigation services as determined in a study of a small community in Vermont (Watson et al. 2016). Insurers and property owners have a vested interest in assessing flood risk and understanding how contributions from riverine wetlands within floodplains influence these determinations.

**Fig. 3:**
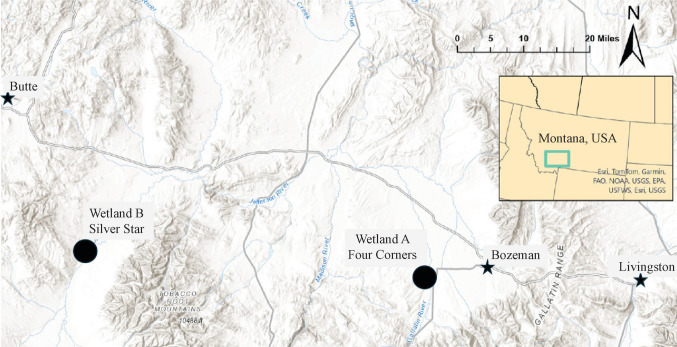
Location of examples wetlands A and B near Bozeman, Montana, USA

**Fig. 4:**
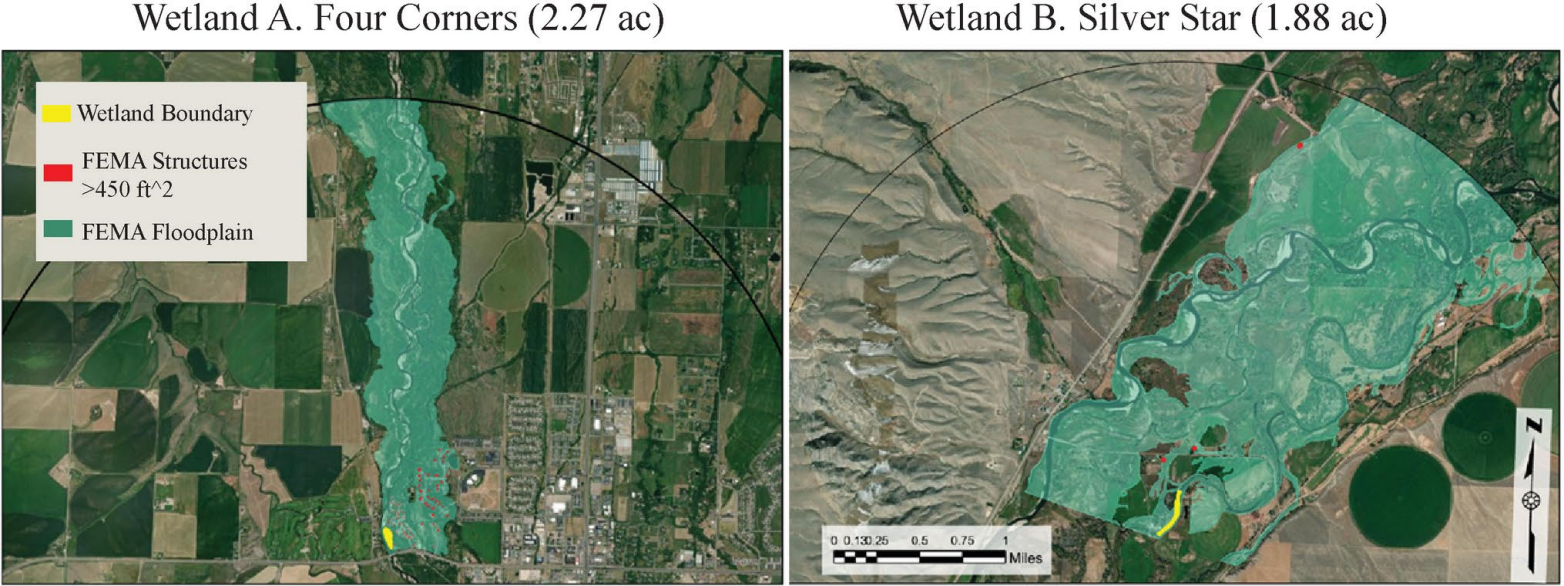
Wetland A – Four Corners is on the left, and Wetland B – Silver Star is on the right. The wetlands are yellow from the National Wetland Inventory (USFWS 2024), the 100-year floodplain is clipped 2.5 miles (4.02 km) downstream of the wetland and is shaded, and the red dots are structures, both from the Federal Emergency Management Administration (FEMA: FEMA 2022)

For our assessment approach, we assume that the wetland’s ability to store surface water dynamically (the wetland’s functional capacity) and its proximity to benefactors who reside within the floodplain (the opportunity) are essential attributes of flood attenuation that benefit a community (Bousquin and Mazzotta 2020; Tang et al. 2020). We use the Montana Wetland Assessment Method (MWAM: Berglund and McEldowney 2008), Montana’s preferred wetland function assessment tool (Kleindl et al. 2024), to assess the wetland function. For this example, we will select two wetlands, Wetland A and Wetland B, located near Bozeman, Montana (Fig. 3). Wetland A, Four Corners, is a 2.27-acre (0.92 ha) floodplain wetland adjacent to the Gallatin River. Wetland B, Silver Star, is a 1.88-acre (0.76 ha) floodplain wetland adjacent to a side channel of the Jefferson River (Fig. 4).

## Assessment

### Wetland Functional Capacity

Like many Tier 2 – rapid assessments, MWAM measures the *capacity* of a wetland to perform a *function* through an FCI (Berglund and McEldowney 2008). MWAM uses two assessment models that address flooding: Flood Attenuation and Short- and Long-Term Surface Water Storage. Referring to our earlier distinctions of assessment language, we believe these titles misrepresent what is measured. Flood Attenuation implies an ecosystem service, but this model only addresses the short-term residence time of in-channel or overbank waters. The model first addresses streambed entrenchment using the Rosgen Stream Classification system (Rosgen 1994). The assumption is that the more entrenched the stream is, the lower the probability of overbank flow, and, therefore, the lower the assessment score. The assessment then focuses on the percentage of flooded wetland area comprised of forested and/or scrub-shrub areas, based on the Cowardin system (Cowardin et al. 1979). This is a surrogate measure of roughness in the wetland, accounting for water residence time during overbank events. The more area covered by forest or shrub, the higher the assessment score. Lastly, the assessment determines if the wetland has an unrestricted, restricted, or no outlet for floodwaters to exit the wetland. Restrictions have a longer residence time. Therefore, no outlet or restricted outlet scores higher than an unrestricted outlet. This model aligns more closely with the Dynamic Surface Water Storage function assessment models in other assessment tools (e.g., Brinson et al. 1995).

The Short- and Long-Term Water Storage assessment model first requires an estimate of the maximum acre-feet of wetland volume that can hold periodic flooding or ponding. The more water a wetland can hold, the higher its assessment score. Next, the duration of surface water inundation is broken into permanent/perennial, seasonal/intermittent, or temporary/ephemeral based on the Cowardin classification system’s water regime modifiers (Cowardin et al. 1979). The longer the water is detained, the higher the assessment score. Finally, the frequency of flooding is determined based on aerial photographs, interviews, or knowledge of the area. The more frequent the flooding, the higher the score. This model aligns more closely with Long-Term Surface Water Storage function assessment models in other assessment tools (e.g., Brinson et al. 1995).

Table 1 shows the results of each attribute and function scores of the two models for Wetlands A and B. The scores are then averaged to account for the functional capacity of the wetlands to capture and hold floodwater. The average of these two models indicates that Wetlands A and B are operating at 40% and 45%, respectively, of what MWAM considers optimal for these two functions (see Berglund and McEldowney 2008). The calculation of the FCI maps to the function assessment results of our conceptual model in Fig. 2

**Table 1:** MWAM floodwater capture and holding attributes and MWAM score the functional capacity for Wetlands A and B's

	Flood attenuation (Dynamic Surface Water Storage)			
Attributes	Rosgen Class	Percent Forested or Scrub/Shrub	Restricted or Unrestricted Outlet	Functional Capacity Index
Wetland A	C Channel	> 25%	Unrestricted	0.50
Wetland B	C Channel	> 25%	Unrestricted	0.50
	Short- and long-term water storage			
Attributes	Estimated ac-ft of storage	Duration of surface water	Frequency of flooding	Functional Capacity Index
Wetland A	~ 1	Seasonal	> 5/10	0.30
Wetland B	~ 1	Perennial	> 5/10	0.40
	Flood holding average functional capacity			
Wetland A				0.40
Wetland B				0.45

### Opportunity

We use our proposed (expanded) definition of *opportunity* by measuring the beneficiaries’ engagement with the products of the wetland processes. Following guidance from USEPA’s Rapid Benefits Indicator approach (RBI) (USEPA 2020b), we created buffers extending to 2.5 miles (4.02 km) downstream from the wetland. We acquired the FEMA flood map (FEMA 2022) and clipped these data to the buffers. FEMA structure database provides data on structures greater than 450 ft^2^ in size, which were counted using FEMA’s structural spatial data (FEMA 2022) and the ArcGIS Near Tool. Wetlands A and B have 101 and 3 structures, respectively, within the FEMA delineated floodplain 2.5 miles (4.02 km) downstream of each wetland. Following our conceptual model in Fig. 2, we define flood mitigation beneficiaries as individuals with structures greater than 450 ft^2^ within this zone.

We use categorical indicators to scale the opportunity for beneficiaries to obtain flood mitigation within the defined zone. Tools such as RBI (USEPA 2020b) have rapidly addressed these attributes using categorical indicators based on the presence or absence of structures within a certain proximity to the wetland assessment area. Florida’s Universal Mitigation Assessment Method (UMAM: Bardi et al. 2004) also uses a categorical array to determine rapid scoring of functions. In our approach, we modified RBI and UMAM to develop an illustrative rapid assessment array of opportunity categories (i.e., Opportunity Array) (Table 2).

**Table 2:** Opportunity array to determine each wetland’s opportunity score (OS) based on the connection of the wetland to the floodplain and the density of structures within the FEMA delineated floodplain 2.5 miles downstream from each wetland. Scoring breaks was guided by protocols used in UMAM (Bardi et al. 2004)

Flood beneficiary opportunity score	Optimal (1.0)	Moderate (0.7)	Minimal (0.4)	Not present (0.0)
Flood Attenuation Benefit Opportunity	Full opportunity to perform flood attenuation service at the optimal level	Partial opportunity to perform flood attenuation service limited to 70% of the optimal level	Partial opportunity to perform flood attenuation service limited to 40% of the optimal level	No opportunity to perform flood attenuation service
a. Is the wetland connected to the FEMA delineated floodplain where people live?	Yes, the wetland is wholly connected to the river and the surrounding floodplain	**-**	**-**	No, the wetland is connected to the river but is wholly disconnected from the floodplain by dikes or other revetments
b. How many structures within the FEMA delineated floodplain benefit from the service within 2.5 miles downstream of the wetland?	There are > = 50 structures within the FEMA flood hazard map	There are > = 25 and < 50 structures within the FEMA flood hazard map	There are > 0 and < 25 structures within the FEMA flood hazard map	There are no structures within the FEMA flood hazard map

In our example, the wetland in Wetland A is fully connected (1.0) to the floodplain and has over 50 structures (1.0); therefore, it would receive an opportunity score of 1.00 (1.0*1.0 = 1.0). Wetland B is also fully connected to the floodplain (1.0); however, there are only three structures downstream of the wetland (0.4), therefore, it receives an opportunity score of 0.40 (1.0*0.4 = 0.4). We use a multiplicative approach here to allow for the possibility of a zero as an opportunity score. For instance, if the wetland were not connected to the floodplain, it would receive a score of 0.0 and would sum to zero regardless of the number of downstream structures because it would not provide any flood attenuation service.

### Wetland Service Capacity and Application of Assessment

We propose that the capacity of a wetland to provide a service is limited by both the capacity of a wetland to provide a function necessary for the service to exist and the opportunity of a community to interact with the products of that function that provide benefits (see Fig. 2). We calculate the SCI for our sample application by multiplying the FCI by the opportunity score (OS), as shown in Table 3. In Wetland A, the SCI is 0.40 (0.40 FCI * 1.00 OS), and for Wetland B, the SCI is 0.18 (0.45 FCI * 0.40 OS). Capacity indices are typically converted to capacity units that act as credits or debits that can be bought or sold to compensate for unavoidable impacts to wetland (33.C.F.R. § 332/40 C.F.R. § 230). In practice, impacts on both functions and services should be compensated through mitigation measures. We follow the methods typical in wetland assessment to calculate capacity units (i.e., Berkowitz et al. 2023). The Functional Capacity and Service Capacity Units (FCU and SCU, respectively) are calculated by multiplying the FCI or SCI by the area of the wetland (Table 3). In this example, the wetlands in Wetlands A and B would provide comparable functions, but Wetland B would provide substantially less services; consequently, the service mitigation credits it would provide would be substantially lower.

**Table 3:** Calculated scores for Wetlands A and B *FCI* Functional Capacity Index, *OS* Opportunity Score, *SCI* Service Capacity Index, *FCU* Functional Capacity Units, and *SCU* Service Capacity Units

Wetland	Area (ac)	Functional capacity index (FCI)	Opportunity score (OS)	Service capacity index (SCI)	Functional capacity units (FCU)	Service capacity units (SCU)
A	2.27	0.40	1.00	0.40	0.91	0.91
B	1.88	0.45	0.40	0.18	0.85	0.34

## Conclusion

For wetland ecosystem service to be effectively incorporated in CWA programs, there needs to be parsimonious assessment tools that can be applied systematically, repeatably, and rapidly. This begins with disentangling existing terminology and clearly defining all the terms necessary to support an ES assessment. The widespread implementation of an ES tool will be most efficient if the tool is designed as a module to be incorporated into existing wetland functional assessment methods. Service Capacity Indices (SCI) fill an important gap in many programs by providing a way to directly evaluate “values,” “services,” “beneficial uses,” or other benefits to humans. These tools can support evaluation of services by incorporated data obtained through ongoing ambient monitoring programs, such as those required under CWA Sect. 305(b), compensatory mitigation needs, such as those required under CWA Sect. 404, or those conducted by the USEPA National Aquatic Resource Survey program, something that is seldom included in current assessment, yet can inform decisions regarding impairment, the need for management intervention, or protection.

Data from ambient monitoring programs may also support dashboards, fact sheets, or other products designed to inform the public about the status of aquatic resources. The application of SCI should be a central element of increasing public awareness about the importance of aquatic resources in their communities. For example, the SCI could be integrated into existing support dashboards designed to inform the public about water resource impairments and communicate how aquatic systems provide beneficial services, such as flood mitigation. Regulatory programs, such as CWA Sect. 404 and 401 permits, compliance with a state’s Coastal Zone Management Act, or state and local wetland permit programs, often require consideration of “ecosystem values or services” in determining impacts, selecting alternatives, and assigning compensatory mitigation. The SCI approach, presented here, provides a way to assess potential impacts on services directly and include those in final permit decisions. Moreover, the SCI can be used to inform the selection, location, and design of mitigation site(s), as well as the associated performance standards, to ensure compensation for permitted impacts to services in addition to functions. Grant-funded voluntary restoration can be utilized to achieve a wide range of ecological and social objectives. To the extent that social needs are prioritized, the SCI can inform the prioritization of sites or projects with the highest probability or greatest potential impact on those needs. As with compensatory mitigation projects, information from the SCI can be used to inform the design and monitoring of voluntary restoration projects.

Overall, the SCI can help bridge gaps in regulatory and monitoring programs by assessing beneficial use opportunities, thus ensuring that wetland services—such as flood mitigation and ecological restoration—are effectively integrated into decision-making and public awareness initiatives. As with existing functional assessment approaches, an SCI approach should be incorporated into repeatable and transparent assessment tools that can be readily and routinely used in existing monitoring and assessment efforts.

## Data Availability

The datasets generated and analyzed during the current study are available from the corresponding author on reasonable request. Initial data were presented in: Kleindl, W. J., S. P. Church, M. C. Rains, and R. Ulrich. 2024. Choosing the Right Tool: A Comparative Study of Wetland Assessment Approaches. Wetlands 44:46.
